# Relationship between the grades of a learned aversive-feeding response and the dopamine contents in *Lymnaea*

**DOI:** 10.1242/bio.021634

**Published:** 2016-11-04

**Authors:** Hitoshi Aonuma, Mugiho Kaneda, Dai Hatakeyama, Takayuki Watanabe, Ken Lukowiak, Etsuro Ito

**Affiliations:** 1 Research Center of Mathematics for Social Creativity, Research Institute for Electronic Science, Hokkaido University, Sapporo 060-0811, Japan; 2 CREST, Japan Science and Technology Agency, Kawaguchi 332-0012, Japan; 3 Laboratory of Functional Biology, Kagawa School of Pharmaceutical Sciences, Tokushima Bunri University, Sanuki 769-2193, Japan; 4 Hotchkiss Brain Institute, University of Calgary, Calgary, Alberta, CanadaT2N 1N4; 5 Department of Biology, Waseda University, Shinjuku, Tokyo 162-8480, Japan

**Keywords:** Conditioned taste aversion, Dopamine, Food deprivation, Long-term memory, *Lymnaea*

## Abstract

The pond snail *Lymnaea* learns conditioned taste aversion (CTA) and remembers not to respond to food substances that initially cause a feeding response. The possible relationship between how well snails learn to follow taste-aversion training and brain dopamine contents is not known. We examined this relationship and found the following: first, snails in the act of eating just before the commencement of CTA training were poor learners and had the highest dopamine contents in the brain; second, snails which had an *ad libitum* access to food, but were not eating just before training, were average learners and had lower dopamine contents; third, snails food-deprived for one day before training were the best learners and had significantly lower contents of dopamine compared to the previous two cohorts. There was a negative correlation between the CTA grades and the brain dopamine contents in these three cohorts. Fourth, snails food-deprived for five days before training were poor learners and had higher dopamine contents. Thus, severe hunger increased the dopamine content in the brain. Because dopamine functions as a reward transmitter, CTA in the severely deprived snails (i.e. the fourth cohort) was thought to be mitigated by a high dopamine content.

## Abstract (precis)

**Summary:** Severely food-deprived snails cannot acquire conditioned taste aversion, because this conditioning is thought to be mitigated by a high content of a reward transmitter, dopamine, in these snails.

## INTRODUCTION

In both the brains of vertebrates and invertebrates, dopamine pathways play an important role in reward-motivated behaviors (Mizunami et al., 2015; Luo and Huang, 2016). In the pond snail *Lymnaea stagnalis*, as well as other molluscs, both pharmacological and immunohistochemical studies of dopaminergic pathways have been conducted (Gospe, 1983; Elekes et al., 1991; Barnes et al., 1994; Kemenes, 1997; Vehovszky et al., 2007). Dopamine has so far been shown to play a key role in appetitive classical conditioning and reward operant conditioning in *Aplysia* (Baxter and Byrne, 2006), as well as in the consolidation of long-term memory (LTM) in appetitive classical conditioning in *Lymnaea* (Kemenes et al., 2011).

Dopamine also plays a role in the neuronal control of feeding in *Lymnaea*. For example, dopamine is present in the buccal ganglion neurons, and its exogenous application activates the feeding response (Elliott and Vehovszky, 2000). Dopamine receptors are located on the cerebral giant cells (CGCs), which modulate feeding behavior (Hernádi et al., 2012). The application of dopamine onto isolated CGC increases their spiking activity (Hernádi et al., 2004). Together, the data suggest that the dopaminergic input alters the activity of the feeding modulatory neuron CGC that in turn should alter feeding behavior.

Conditioned taste aversion (CTA) is formed following training where we pair presentation of a sucrose solution (the conditioned stimulus; CS) and an electric shock (the unconditioned stimulus; US) (Ito et al., 2013; Takigami et al., 2013). In snails, CTA persists for at least one month (Kojima et al., 2015). Previously, we demonstrated that the CGCs play a key role in CTA (Yamanaka et al., 1999; Kojima et al., 2001; Otsuka et al., 2013). Here we (1) measured dopamine contents in the brains of *Lymnaea* subjected to different periods of food deprivation (Yamagishi et al., 2015); and (2) correlated how good CTA was compared to the contents of dopamine in their brains.

In addition, we also measured the 3,4-dihydroxyphenylalanine (l-DOPA) and 3,4-dihydroxyphenylacetic acid (DOPAC) content of the brains simultaneously. l-DOPA is the precursor molecule of dopamine, and it is converted to dopamine by l-aromatic amino acid decarboxylase at rates so rapid that DOPA contents in the mammalian brain are negligible under normal conditions (Cooper et al., 2003). DOPAC is a catabolite of dopamine, and dopamine is converted to DOPAC by monoamine oxidase (MAO) after reuptake by the nerve terminal. Although dopamine is also converted to homovanillic acid (HVA) through the sequential action of catechol-*O*-methyltransferase (COMT) and MAO (Cooper et al., 2003), here we focused only on DOPAC but not HVA.

## RESULTS

### CTA grades

We used four cohorts of naive snails for the CTA training: (A) snails that were in the act of eating just before the commencement of the CTA training procedure (i.e. Eating snails); (B) snails that had been given *ad libitum* access to food but were not eating just prior to the CTA training procedure (called Day −1 snails); (C) snails that had been food-deprived for 1 day (called Day 1 snails, i.e. moderately hungry snails), that is, Day −1 snails could eat freely before the start of CTA training, whereas Day 1 snails experienced food deprivation at least 1 day before the training; and (D) snails that had been food-deprived for 5 days (Day 5 snails, i.e. severely hungry snails) (Mita et al., 2014a,b).

In the pre- and various post-test sessions (10 min, 1 h, 1 day, and 1 week) given to all four cohorts of snails, we counted the number of bites in response to the CS (sucrose) for 1 min. In all four cohorts of snails, we performed two controls, backward conditioning and naive training; and we found no significant decrease in the feeding response to the CS in these control groups.

**Fig. 1.**
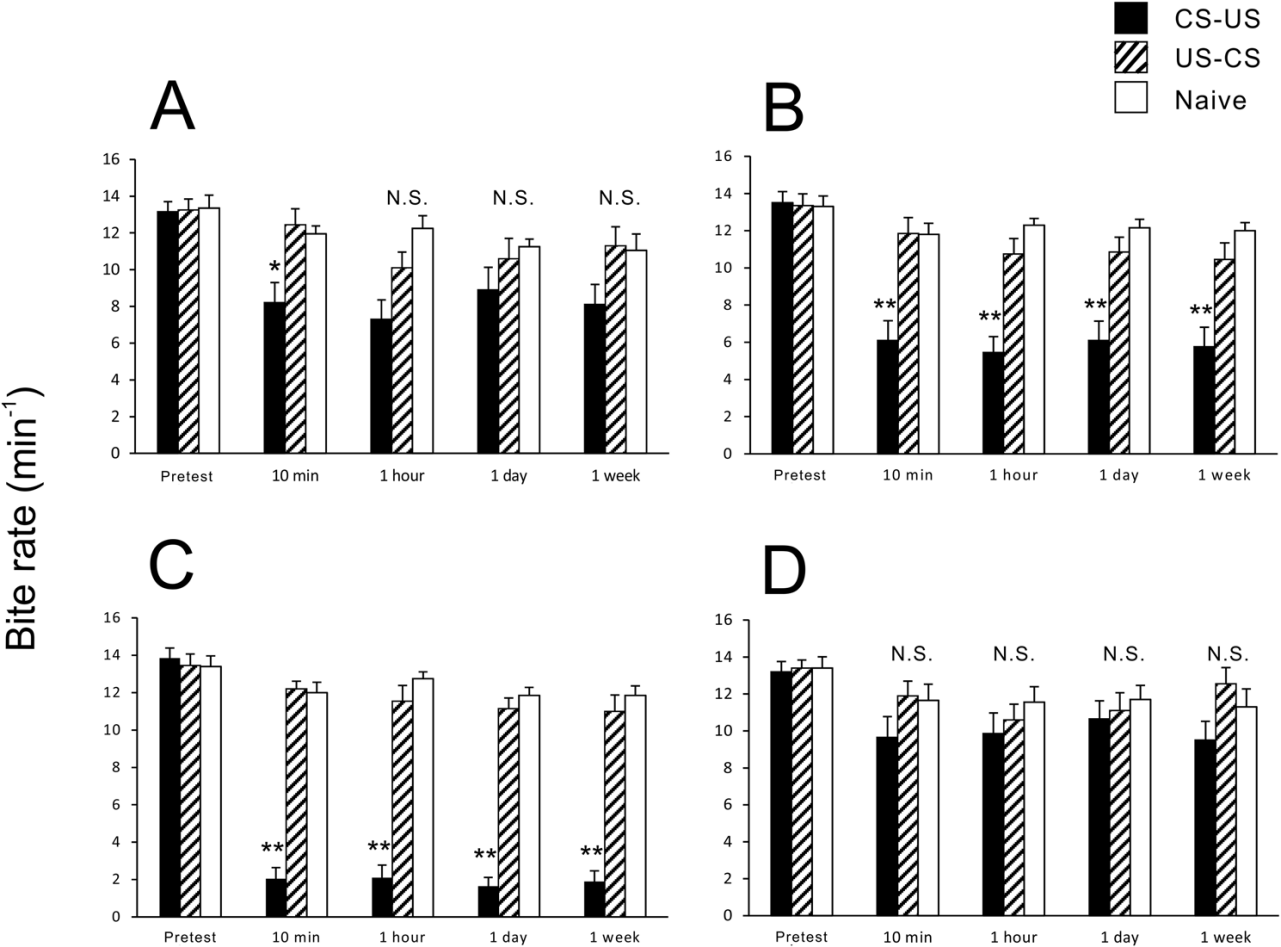
**Grades of CTA learning and memory under various starvation states.** We tested CTA trained snails (black bars), backward-conditioned snails (hatched bars) and naive control snails (white bars). *N*=20 each. (A) Eating snails. After 20 pairings of the CS and the US, the feeding responses were weakly suppressed in comparison with those of control snails (fair grade, *F*_14,285_=4.818, *P*<0.01), and CTA memory persisted for at least one week (*P*<0.05). (B) Day −1 snails. The feeding responses were suppressed (good grade, *F*_14,285_=14.158, *P*<0.01), and CTA memory persisted for at least one week (*P*<0.01). (C) Day 1 snails. The feeding responses were strongly suppressed (excellent grade, *F*_14,285_=63.700, *P*<0.01), and CTA memory persisted for at least one week (*P*<0.01). (D) Day 5 snails. The feeding responses were not suppressed (poor grade, *F*_14,285_=2.064, *P*>0.05), and CTA memory was not formed (*P*>0.05). * indicates *P*<0.05; ** indicates *P*<0.01. *P* values were determined by one-way ANOVA and post hoc Tukey test. Error bars indicate s.e.m.

We found that the state of eating before training had a significant impact on how well or how poorly the snails learned and remembered. CTA-trained snails that were in the act of eating (Eating snails) showed learning but not memory formation (Fig. 1A). That is, while the number of bites in the 10-min test session after training was significantly reduced (*P*<0.05), the number of bites 1 h, 1 day and 1 week later were not different from the pre-training levels (*P*>0.05). In this cohort of snails, the feeding ratio between the result of the pretest and that of the 10-min post-test was determined to be 61%.

The next cohort of snails tested was the Day −1 cohort (while these snails had food *ad libitum*, they were not eating just before training). In these snails, following training the response to the CS was significantly suppressed at the 10-min post-test (*P*<0.01, Fig. 1B), and the feeding response continued to be suppressed at the 1 h and 1 week memory test sessions (*P*<0.01). Thus, the snails in this cohort learned and formed memory. When we determined the feeding ratio of this cohort, it was found to be 45%.

We examined how well snails performed following 1 day of food deprivation before training (Day 1 snails). We found that these snails exhibited excellent learning and memory formation in all the post-training sessions (*P*<0.01, Fig. 1C). When we determined the feeding ratio of this cohort, it was found to be 15%. In the final cohort training after 5 days of food deprivation (i.e. the Day 5 snails), the feeding response elicited by the CS in all the post-test sessions was not significantly different from those in the control snails in the pretest (*P*>0.05, Fig. 1D). Thus, learning and memory formation did not occur in these snails.

We conclude that the state of the snail as regards its eating history, or lack thereof, significantly alters how well or how poorly they learn and form memory following CTA training.

### Dopamine contents in the brain

Having obtained the behavioral data from the four cohorts, we then measured the dopamine content of their brains using high-performance liquid chromatography with an electrochemical detection system (HPLC-ECD). The dopamine content of the Eating snails, which were eating just before training, was higher than the snails that had access to food but were not eating (i.e. Day −1 snails). Further, the dopamine content of Day −1 snails was higher than the snails that were food-deprived for one day (i.e. the moderately hungry state; Day 1 snails). We then compared the behavioral grades earned by these three cohorts (i.e. Eating snails, Day −1 snails and Day 1 snails) with their respective dopamine contents (Fig. 1), and found that the better the grade, the lower the dopamine content in the brain. Thus, there was a negative correlation between the assessed grade and the dopamine content in the brain.

Interestingly, the dopamine content of the snails that were food-deprived for 5 days (i.e. the severely hungry state; Day 5 snails) was significantly higher (*P*<0.05) than that of the Day 1 cohort, and these Day 5 snails were the poorest learners (Fig. 1). CTA in the severely deprived snails was thought to be mitigated by a high dopamine content.

**Fig. 2.**
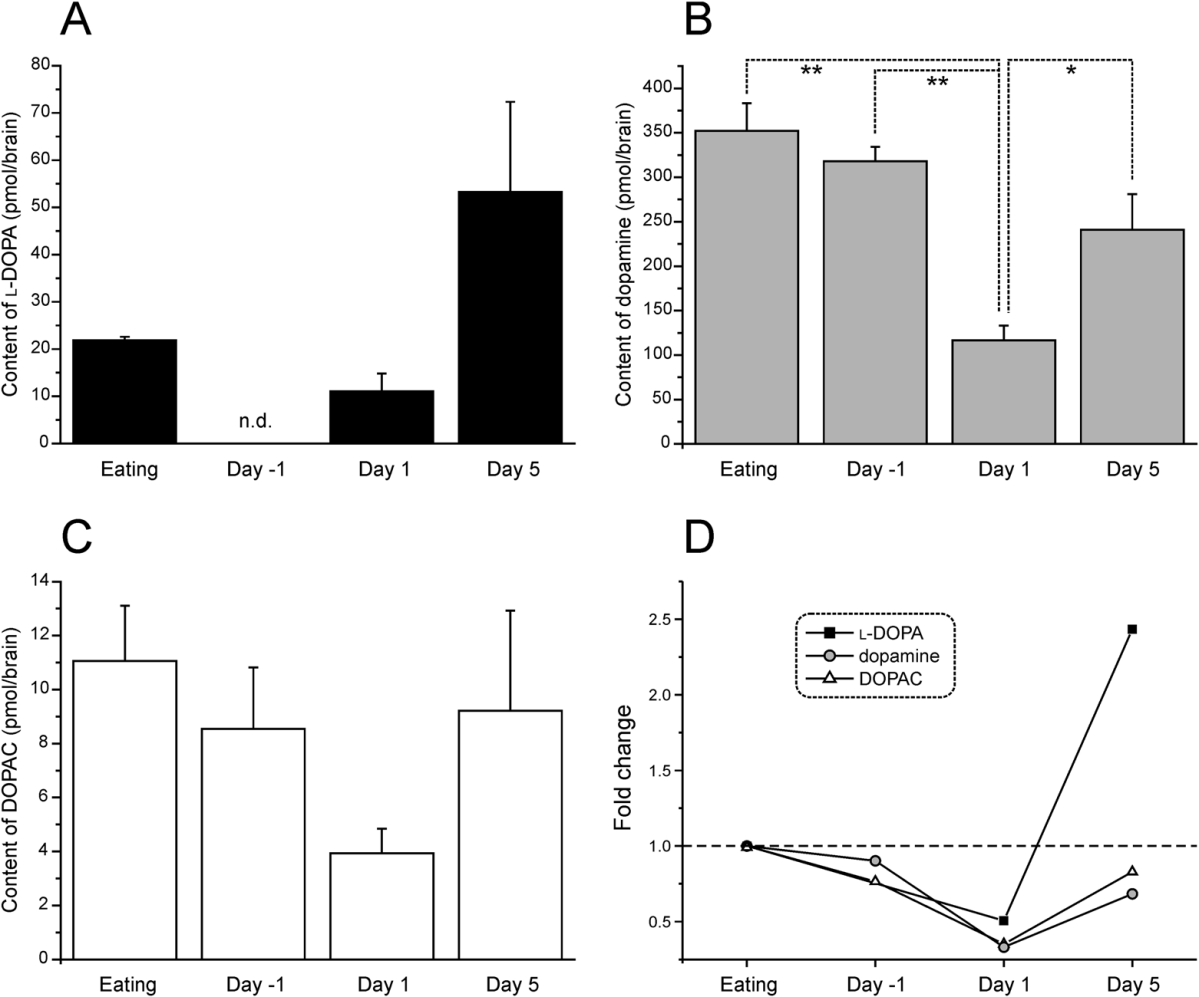
**Changes in contents of l-DOPA, dopamine and DOPAC in the brain isolated from snails with various starvation states.** (A) l-DOPA. *F*_2,6_=4.223, *P*>0.05. (B) Dopamine. *F*_3,35_=12.672, *P*<0.01. **P<0.01 and *P<0.05; one-way ANOVA and post hoc Scheffe test. (C) DOPAC. *F*_3,22_=2.591, *P*>0.05. (D) The data were normalized by that of Eating snails. The contents of l-DOPA, dopamine and DOPAC in Day 5 snails were higher than those in Day 1 snails. We failed to measure the content of l-DOPA in Day −1 snails. Dashed line indicates the base value of Eating snails.

In the four cohorts of snails (Eating, Day −1, Day 1 and Day 5 snails) the contents of l-DOPA and DOPAC were measured (Fig. 2A). To easily understand the changes, the mean values of the data were normalized to that of the Eating snails (Fig. 2B). We could not measure the content of l-DOPA in Day −1 snails due to technical difficulties. There was a similar correlation in these two contents with the state of the snails with regards to their feeding history, as well as the case of dopamine. That is, in the more starved snails, there was a lesser amount of these molecules. However, in Day 5 snails, the contents of l-DOPA and DOPAC were higher than those in Day 1 snails, as well as the case of dopamine.

## DISCUSSION

In molluscs, dopamine plays an important role in appetitive classical conditioning (Baxter and Byrne, 2006; Kemenes et al., 2011). Here we found that dopamine contents in the brains of snails given CTA training correlate with how well snails learn and remember, as expressed by the calculated feeding ratio. In this case, there is a negative correlation as the better the learning the lower the dopamine content of the brains. Because dopamine is sometimes considered as a reward transmitter (Mizunami et al., 2015; Luo and Huang, 2016), it follows then that dopamine contents should be lower in the ‘good’ learners as the CS is no longer viewed by the snail as rewarding. That is, following learning and memory formation of CTA, the CS now signals punishment rather than reward. We hypothesize that as part of the changes taking place in the brain with successful CTA training and subsequent memory formation, there must be a decrease in dopamine contents. We are uncertain how dopamine contents are normally managed, nor do we know how training alters the contents; however, we do understand why the dopamine contents in Day 5 snails are so high.

In the present study, we cannot claim that there is causality between the CTA grades and the dopamine contents in the brain, especially for the data obtained from Day 5 snails. However, we need to keep in mind the following:

(1) Food deprivation suppresses the egg-laying behavior in *Lymnaea* (Ter Maat et al., 1982). The egg-laying behavior is partly controlled by monoamines including dopamine (Boyle and Yoshino, 2002). That is, food deprivation is thought to have some effects on dopamine contents in *Lymnaea*, and so it seems reasonable that there is a negative correlation between the CTA grades and the brain dopamine contents in Eating, Day −1 and Day 1 snails.

(2) The Day 5 snails received both the poorest grade and had the highest dopamine contents (Fig. 1). It turns out, however, that while they do not exhibit the behavioral memory phenotype, there is CTA memory in these snails but the memory is occluded. That is, there is a conflict between the CTA memory and the desire/necessity to eat. As described in an earlier report (Ito et al., 2015b), the ‘necessity knows no law’ concept prevails in these snails. That is, snails do not express the memory phenotype if they are extremely hungry (for example, Day 5 snails), i.e. CTA memory was formed but is overwhelmed by hunger. This could be shown because the memory is context-dependent (Haney and Lukowiak, 2001; Lukowiak et al., 2007) and can only be observed when the snails are in a context similar to that in which the training occurred. Thus, if the Day 5 snails are fed after they received the training, the memory phenotype can be expressed (Ito et al., 2015b). Recently, dopamine was also shown to be involved in positive emotion states, even in invertebrates (Perry et al., 2016). Our present findings thus suggest that one of the factors contributing to the ‘decision’ or ‘emotion’ to eat or not to eat is the dopamine content in the brain.

We also know that there is a possible interaction between insulin and dopamine in the expression of CTA memory. We found previously that following the injection of insulin into Day 5 snails, CTA was observed (Mita et al., 2014b). We are currently performing experiments looking at how insulin may alter dopamine contents, as insulin plays a major role through its interaction with transcription factors in CTA memory formation (Nakamura et al., 1999; Hatakeyama et al., 2006; Murakami et al., 2013).

**Fig. 3.**
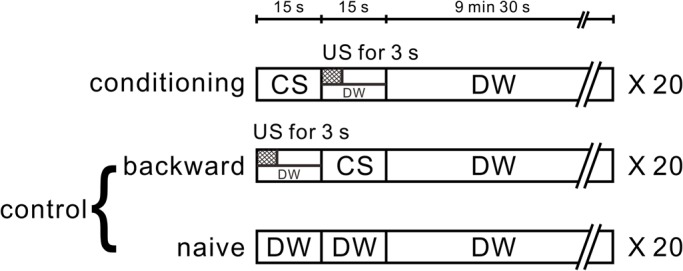
**Learning procedure for conditioned taste aversion in *Lymnaea*.** All snails were first given a pretest. In this observation period (1 min), the number of feeding responses (i.e. bites/min) was counted in distilled water following a 15-s application of 10 mM sucrose (the CS) to the lips of the snail. For CTA training, the 10 mM sucrose CS was paired with a 3-s high voltage electric shock (the US). Following the 3-s electric shock, a 12-s recovery period was required in the US period. The inter-stimulus interval was 15 s between the onset of the CS and US. A 10-min inter-trial interval was interposed between each pairing of the CS-US. Snails received 20 paired CS-US trials on a single day. Controls included a backward-conditioned (US-CS) group and a naive group to validate associative learning. For the naive control group, only distilled water was applied to the lips instead of the CS and US. In the post-test sessions, snails were again challenged with the CS, and the number of bites was recorded in the 1-min interval in distilled water after a 15-s application of the CS.

As with dopamine contents in severely hungry (Day 5) snails, the l-DOPA content was significantly higher than those of dopamine and DOPAC (Fig. 3). l-DOPA is generally known as a precursor of dopamine, but also exhibits neurotransmitter-like actions in mammals (Yue et al., 1993; Misu et al., 2002, 2003). A similar action has been reported in regards to the RPeD1 neuron in *Lymnaea*. Exposure of the RPeD1 neuron to l-DOPA caused a biphasic effect composed of a depolarization followed by a hyperpolarization (Dyakonova et al., 2009). Recently, orphan G-protein coupled receptor (OA1) was identified as a receptor of l-DOPA in mammals (Lopez et al., 2008). In *Lymnaea*, four related genes encoding a family of orphan G-protein coupled receptors have been isolated, and mRNAs of these receptors were transcribed in the CGCs, which are interneurons to control a central pattern generator of fictive feeding behavior (Saunders et al., 2000). Additionally, in *Xenopus* oocytes expressing mammalian AMPA-type glutamate receptor, l-DOPA induced small inward currents which were abolished by kynurenic acid and CNQX (Miyamae et al., 1999), suggesting that l-DOPA can activate ionotropic glutamate receptor. Neurons, containing CGCs and the buccal motor neurons that control muscles involved in the feeding response, respond to bath application of glutamate (Nesic et al., 1996). Taken together, in severely hungry snails (i.e. Day 5 snails), l-DOPA is suggested to control and/or modulate feeding behavior of *Lymnaea* via the so-called orphan G-protein coupled receptors and ionotropic glutamate receptors.

In the present study, we have focused only on the contents of dopamine and its related catabolites in *Lymnaea* with various eating histories. In the future, we will also pay attention to the serotonin contents, as they are also affected by eating history (Elliott and Vehovszky, 2000; Hernádi et al., 2004). During food intake, the external and internal food stimuli are thought to increase the activity of the central monoaminergic system, and also to increase the contents of monoamines (i.e. dopamine and serotonin) in the *Lymnaea* brain. Further, it was also suggested that the increased dopamine and serotonin contents both affect the activity of the serotonergic neurons during the different phases of feeding (Hernádi et al., 2004).

## MATERIALS AND METHODS

### CTA training procedure

Specimens of *Lymnaea stagnalis* (Linnaeus, 1758) with an 18-23 mm shell obtained from our snail-rearing facility (original stocks from Vrije Universiteit Amsterdam) were used in the present study (Kojima et al., 1998). All snails were maintained in dechlorinated tap water (i.e. pond water) under a 12 h:12 h light-dark cycle at 20°C and fed *ad libitum* on turnip leaves [*Brassica rapa* var. *perviridis*; Komatsuna (in Japanese)] and a commercially available product called ‘spiral shell food’ (a combination of seaweed, brewer's yeast and vitamins; Nisso, Saitama, Japan) every other day. *Lymnaea* exhibit good growth and reproduction under these conditions (Ito et al., 1999).

The basic procedure of CTA training has been previously described (Fig. 3) (Sugai et al., 2006). Briefly, all snails were first given a pretest in polystyrene petri dishes (diameter 35 mm). That is, we counted by visual inspection the number of feeding responses (i.e. bites) elicited by the CS (10 mM sucrose solution) in the 1-min period after presentation of the CS. 1 ml of CS was gently applied to the lips of snails with a pipette. Following the pretest, the snails were subjected to the conditioning and control procedures in the same petri dish as used for the pretest. In the CTA training procedure, we paired the CS with the US (3-s electric shock) (Ito et al., 2015a). This US was a high voltage electric shock of minimal current with distilled water (Takigami et al., 2013; Mita et al., 2014b), and was applied near the head in the distilled water. We did not touch the head with the stimulator. Thus, small variations in distance between the snails and the electrode had minimal effect on the current delivered to the snail. The stimuli were required at a level to evoke the withdrawal response. The CS was rinsed out by distilled water and then followed by the US. The US period was set as 15 s, because following the 3-s electric shock a 12-s recovery time was needed for the body to re-emerge from the shells (Ito et al., 2015a). Snails received 20 paired presentations of the CS-US. Controls included a backward-conditioning (US-CS) group and a naive group to validate associative learning. For the naive control group, only distilled water was applied to the lips instead of the CS and US. The number of snails used was 20 for each group. In the post-test sessions, snails were again challenged with the CS, and the number of bites was recorded in the 1-min interval in distilled water after a 15-s application of the CS. The post-tests were performed 10 min, 1 h, 1 day and 1 week after training. In all experiments, after the 10-min post-test, snails were allowed *ad libitum* access to food. All tests were performed blind. The behavioral experiments were performed in the morning because it has been shown that the learning scores are better in the morning than at other times (Wagatsuma et al., 2004).

The suppression ratio of feeding response after CTA training was calculated as follows. The mean value of number of bites elicited by the CS in the 10-min post-test was divided by that in the pretest, and then we expressed these values as a percentage.

### Measurement of dopamine content in the brain

The measurement method was as described previously (Aonuma and Watanabe, 2012), with a small modification. Snails were quickly frozen using liquid N_2_, and the brain was dissected out in the ice-cold saline. Each brain was then homogenized in 50 µl of ice-cold 0.1 M perchloric acid containing 5 ng of N-ω-methyl-5-hydroxytryptamine oxalate (NMET; Sigma-Aldrich, St. Louis, MO, USA) as an internal standard. After centrifugation of the homogenate [0°C, 21,500 ***g*** (15,000 rpm), 30 min], 40 µl of supernatant was collected. Dopamine, l-DOPA and DOPAC in the single brain were measured using high-performance liquid chromatography (HPLC) with electrochemical detection (ECD; EICOM, Kyoto, Japan). The number of single brains used was 10 for each group. The brain included all ganglia. The mobile phase containing 0.18 M chloroacetic acid and 16 µM disodium EDTA was adjusted to pH 3.6 with NaOH. Sodium-1-octanesulfonate at 1.85 mM as an ion-pair reagent and CH_3_CN at 8.40% (v/v) as an organic modifier were added to the mobile phase solution. The chromatographs were acquired using the computer program PowerChrom (eDAQ Pty, Denistone East, NSW, Australia). The supernatants of samples were injected directly onto the HPLC column. The snails used for the measurement of dopamine were independent from those used for behavioral experiments to avoid the effects of sucrose application on the dopamine contents.

### Data analysis

Data are expressed as the mean±s.e.m. Differences in the contents were tested using one-way ANOVA and post hoc Tukey test or Scheffé test (*P*<0.05).
